# Randomised Controlled Trial of an Online Version of Compassion Mind Training in a Nonclinical Sample

**DOI:** 10.5964/ejop.v16i2.1683

**Published:** 2020-05-29

**Authors:** Júlia Halamová, Martin Kanovský, Alexandra Pačutová, Nuriye Kupeli

**Affiliations:** a Institute of Applied Psychology, Faculty of Social and Economic Sciences, Comenius University in Bratislava, Bratislava, Slovakia; b Institute of Social Anthropology, Faculty of Social and Economic Sciences, Comenius University in Bratislava, Bratislava, Slovakia; c Marie Curie Palliative Care Research Department, Division of Psychiatry, University College London, London, United Kingdom; Department of Psychology and Counselling, Webster University Geneva, Geneva, Switzerland

**Keywords:** self-criticism, self-compassion, Compassionate Mind Training, Randomized Controlled Trial, experiment

## Abstract

Compassion Mind Training (CMT) is a therapeutic approach to guide highly self-critical individuals to generate compassion. The goal was to probe the efficacy of a short-term, online version of the CMT on self-compassion and self-criticism in a non-clinical population. We conducted a randomized controlled trial with pre-, post-measurements and two-month follow-up. Out of 144 randomly allocated participants 26 and 20 of those allocated to the intervention and control groups, respectively, completed the follow-up measures. The intervention group was instructed through email to practice a different CMT exercise every day for 13 consecutive days. There was a significant effect of the intervention on self-criticism, especially Hated-self and the Self-uncompassionate responding. The CMT group reported a reduction in negative thoughts and feelings with effects present at the two-month follow-up. There was no significant effect of the intervention on self-reassurance and self-compassion. Self-criticism is amenable to change following a short-term online intervention of CMT delivered to a non-clinical population with effects lasting at least two months. These findings are promising and suggest that interventions designed to reduce self-criticism can be provided to broader populations without direct involvement of mental health professionals.

Evidence suggests that self-criticism contributes to the development and maintenance of psychopathology (Shahar et al., 2012), including anxiety, depression, post-traumatic stress disorder, alcoholism, self-harming behaviours, suicidal tendencies, bipolar disorder, schizophrenia, eating disorders, and borderline personality disorder. Similarly, recent meta-analyses have identified a causal inﬂuence of self-compassion on well-being (Zessin, Dickhäuser, & Garbade, 2015) and an inverse relationship between self-compassion and psychopathology (MacBeth & Gumley, 2012). According to Gilbert and Irons (2004), self-criticism is an essential focus for intervention in psychotherapy and can be treated by learning compassion and self-compassion. This is particularly important as research suggests that self-critical people have difficulties with self-compassion, self-soothing and self-warmth (Gilbert, 2010b).

## Compassion-Focused Therapy (CFT) and Compassion Mind Training (CMT)

To date, Compassion-Focused Therapy (CFT) (Gilbert, 2010b) is one of the most evaluated compassion-based intervention programs (Kirby, 2017). In a systematic review of 14 studies, Leaviss and Uttley (2015) found evidence of the effectiveness of CFT as a psychotherapeutic intervention across clinical and nonclinical samples. However, for CFT to be considered evidence-based practice, large-scale and high-quality trials are needed (Leaviss & Uttley, 2015).

The main goal of CFT (Gilbert, 2010b) is to harmonize and balance the following three emotion regulation systems: threat/ protection, drive/reward, and affiliation/soothing systems. The essential psychotherapeutic technique of CFT is Compassionate Mind Training (CMT), by which clients are taught the skills of compassion and self-compassion (Gilbert, 2010a) and to be able to self-soothe at times of difficulty by improving accessibility to the affiliation-focused affect system.

## The Impact of CMT on Self-Compassion and Self-Criticism

A review of the studies examining the impact of CMT on self-compassion and self-criticism was conducted. The inclusion criteria for the present review were studies which have: (1) used CMT as an intervention or as part of a program, (2) recruited at least 5 participants, and (3) used quantitative measures of self-criticism and/or self-compassion.

In summary, 10 studies have examined the impact of CMT on self-criticism and self-compassion (see Appendix 1 for a summary). All but two (Beaumont, Irons, Rayner, & Dagnall, 2016; Matos et al., 2017) of the studies have recruited clinical samples and all but two (Matos et al., 2017; Noorbala et al., 2013) of the studies were conducted in the United Kingdom and thus published in English. Noorbala, Borjali, Ahmadian-Attari, and Noorbala (2013) delivered CMT to a sample of patients with Major Depressive Disorder in Iran and Matos and colleagues (2017) examined the psychological and physiological effects of the CMT in a nonclinical sample in Portugal. Matos et al. (2017) found that the experimental group reported significant increases in self-compassion, and a reduction in self-criticism. This group also experienced significant improvements in heart rate variability suggesting that CMT has positive influences on both perceived and physiological outcomes.

However, this review also demonstrated some inconclusive findings. For example, CMT is suggested to influence changes in self-criticism (Gilbert & Procter, 2006) or self-compassion (Gilbert & Irons, 2004), whilst others found that CMT to impact both self-compassion and self-criticism (Judge, Cleghorn, McEwan, & Gilbert, 2012; Matos et al., 2017).

A similar format of delivering CMT was used in majority of the studies. The intervention lasted approximately 6 to 12 weeks, with each session lasting between 1-2 hours, and was delivered in group meetings once or twice a week. However, Matos et al. (2017) explored the impact of group-based CMT delivered over a shorter two-week period. Although Zettle, Haflich, and Reynolds (1992) originally found that self-critical people respond better to individual therapy compared to those who were assigned to group therapy, the majority of studies on CMT are in group format.

To summarise, Zessin, Dickhäuser, and Garbade (2015) suggested that a variety of interventions have been developed to increase self-compassion in the long term, but limited research has explored the short-term effects (Krieger, Martig, van den Brink, & Berger, 2016; Matos et al., 2017). More importantly, it is well known that approximately two-thirds of people with mental health problems do not seek help from a health professional (WHO, 2001). Therefore, it is essential to develop interventions which are accessible for a multitude of people. An online method of administering a CMT intervention will overcome the time and financial costs associated with psychological treatments and minimize the possible instillation of social stigma associated with direct contact with health professionals in interventions delivered in a group format. Thus, the aim of this study was to explore the impact of an individual short-form version of CMT delivered through an online setting to be completed in the privacy of ones’ own home.

## Aim

In the current research study, our goal was to evaluate the impact of an online and abbreviated version of CMT in a 13-day version on self-compassion and self-criticism in a non-clinical population.

## Method

### Participants

Convenience sampling was used to recruit participants from the general community online through various social media and social networking sites. To increase motivation to participate, those who completed all three measurements of the study were entered into a prize draw to win a tablet (Halamová, Kanovský, Varšová, & Kupeli, 2018). 

**Figure 1:**
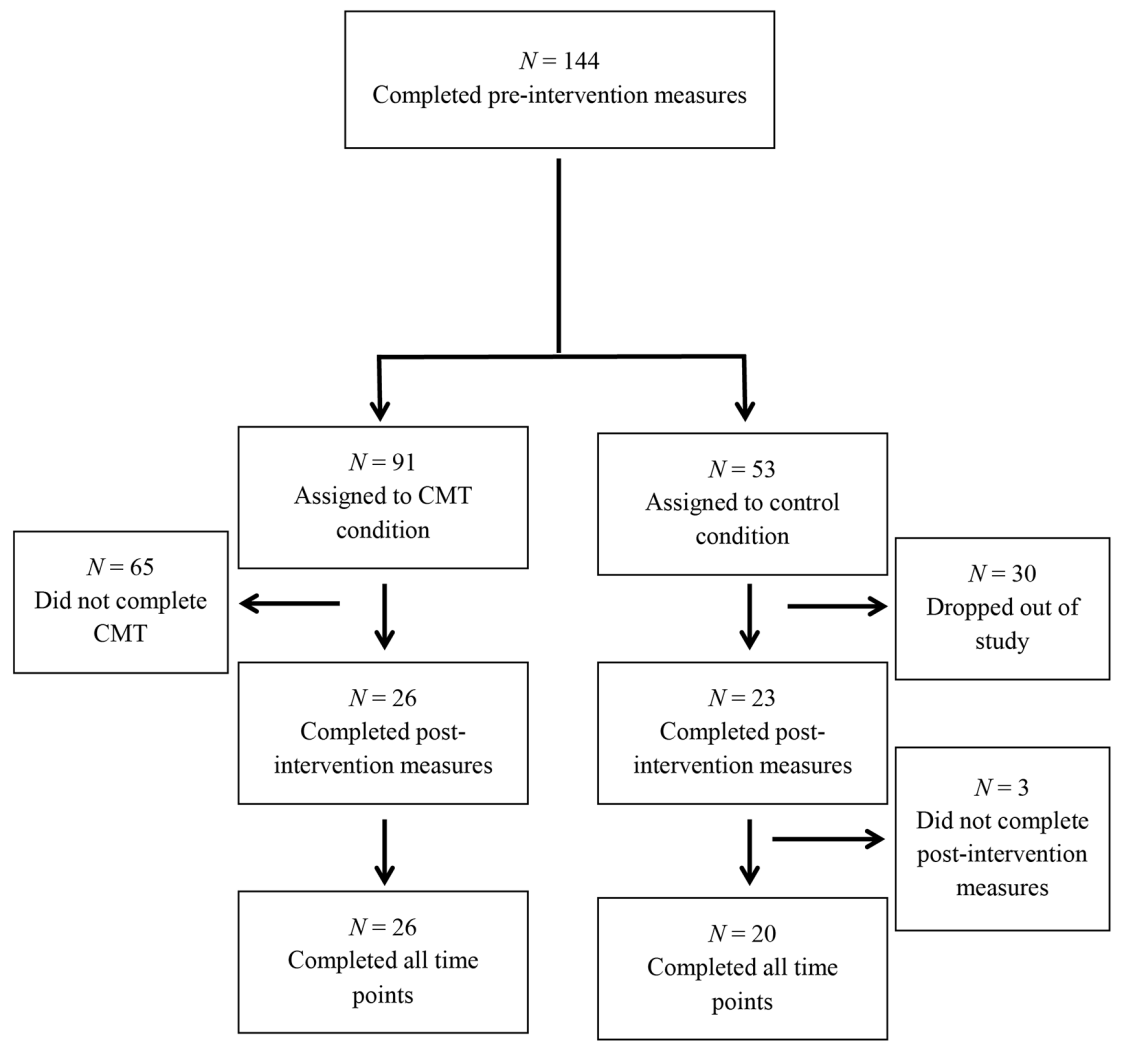
Flow chart for the number of participants who completed each stage of the study and attrition.

The participants were part of a larger RCT examining the impact of four different compassion-based interventions on reducing self-criticism and increasing self-compassion (results of the other compassion-based interventions are published elsewhere e.g. Halamová, Kanovský, Varšová, & Kupeli, 2018). The focus of this paper is the CMT intervention and for this a total of 144 participants completed the pre-intervention measures of which 91 participants were randomly allocated to the CMT group and 53 were randomly assigned to the control condition (Halamová, Kanovský, Varšová, & Kupeli, 2018). See Figure 1. Due to the level of commitment required for the intervention, we expected a higher dropout rate in this group compared with the control group. Therefore we used a block randomization procedure to assign the first 15 participants to the intervention group followed by allocating the next eight participants to the control group until all participants were allocated to the groups. From this sample, 26 from the intervention group and 23 participants from the control group completed the post-intervention. A final sample of 46 participants completed the follow-up measures of which 26 participants completed the CMT task and 20 participants were assigned to the control group. The final intervention group consisted of 22 women and 4 men with mean age of 26.23 years (*SD* = 6.43) and the control group consisted of 17 women and 3 men with a mean age of 25.35 years (*SD* = 6.32).

### Instruments

Self-criticism/reassurance was assessed using the *Forms of Self-Criticism/Reassuring Scale* (FSCRS; Gilbert, Clarke, Hempel, Miles, & Irons, 2004). The FSCRS is a 22-item self-report measure requiring participants to rate a selection of positive and negative statements on a 5-point Likert scale (“Not at all like me” to “Extremely like me”). Items include “I am easily disappointed with myself” and “I am gentle and supportive with myself”. Positive items reflect the ability to self-reassure (referred to as reassured-self [RS]) and negative items indicate self-critical thoughts and feelings (split into subscales of inadequate-self [IS] and hated-self [HS]). This scale has been translated and validated across a number of cultures (e.g. Halamová, Kanovský, & Pacúchová, 2017; Halamová, Kanovský, Gilbert et al., 2018; Petrocchi & Couyoumdjian, 2016).

The *Self-Compassion Scale* (SCS; Neff, 2003) was used to measure self-compassion experienced during perceived difficulty. The SCS comprises six components of self-compassion that measure kindness towards the self rather than critical self-judgement (similar to the constructs of self-reassurance and self-criticism, respectively), common humanity in which it is accepted that failure and pain are part of the human experience rather than a perceived isolation from this human experience, and mindful awareness of negative emotional states in which these are faced and accepted rather than denied or exaggerated as in over-identification of emotional states (Neff, 2003). The scale consists of 26 items rated on a 5-point Likert scale (“Almost never” to “Almost always). The SCS has been validated and demonstrated that positively formulated items (self-judgement, isolation and over-identification) and negatively formulated items (self-kindness, common humanity and mindfulness) SCS subscales are recommended to be calculated separately (e.g. Brenner, Heath, Vogel, & Credé, 2017; Halamová, Kanovský, & Pacúchová, 2018; López et al., 2015) even across different cultures (Halamová, Kanovský, Petrocchi et al., 2020).

### Procedure

After completing sociodemographic information and the first (baseline) measurement, all were randomly allocated to the intervention group and control group. The control group did not receive any additional tasks until 13 days after completion of the baseline measures when they were asked again by an email to fill out the online self-report measures. Again after two months they were asked to fill it out. 

Participants allocated to the online intervention were required to accomplish a CMT exercise every day for 13 consecutive days which commenced the day after they were allocated to the condition. Each participant received an email prompting them to complete the CMT task by following a link. Each exercise was presented in the same format and order for each participant. Following our experience of conducting interventions, we applied a simple formula: explain what the task is, what the potential impact of task could be for health why people should do it, let them do it and then reflect on the task to increase the potential impact of the exercise. The post-exercise questions required free-text responses to items such as “What aspect of the exercise will you use in your everyday life and how?” or “Describe your safe place”. The additional function of the post-exercise task was a fidelity check. If the participant had not completed the exercise, they were sent an email reminder. The exercises were selected from a multitude of different exercises available in Compassionate Mind Training (Gilbert, 2010a). Each exercise was included after reaching consensus of our research team based on representativeness of CMT and the expected motivation of participants to complete them. The first author of this article is an experienced psychotherapist trained in Introduction to Compassion-Focused therapy and Advanced Clinical Skills Workshop in Compassion-Focused therapy. The exercises were translated and shortened by two of the authors (AP and JH). Participants were able to access the intervention tasks on any computer or smartphone via a link on the day the email was sent. The exercises (see Appendix 2 for a summary) selected and presented to participants in the following order were, “Soothing Rhythm Breathing”, “Compassionate Body Scan”, “Imaging the Self-Critical part of Self”, “Creating a Safe Place”, “Compassionate Colour”, “Developing the Inner Compassionate Self”, “Working with Troubled Self”, “Working with Anxious Self”, “Compassion Flowing into you from Others”, “Compassion Flowing Out”, “Compassionate Letter Writing”, “Compassionate Dialogue Writing”, and "Creating your Ideal Caring-compassionate Image". Following the final exercise, participants were sent an email with a link to the post-intervention (second) measures and again at the two-month follow-up (third measures).

### Data Analyses

The statistical program R (Version 3.4.0; R Core Team, 2013) was used for statistical analyses, library nparLD (Noguchi, Gel, Brunner, & Konietsche, 2012). We admit that parametric analyses (ANOVA) are commonly used for factorial research designs of our type (i. e. split-plot design). However, parametric ANOVA analyses require strict assumptions to be met: normal distribution, equal error variances, sphericity, and absence of outliers. It is known that their non-parametric counterparts (Kruskal-Wallis test and, Wald statistics) have poor performance for small sample sizes and designs unbalanced in terms of equal sample size of groups; see Brunner, Domhof, & Langer, 2002; Brunner, Konietschke, Pauly, & Puri, 2017; Brunner, Munzel, & Puri, 1999). In our data, the outcome variables are sum scores of categorial (ordinal) item scores, therefore we cannot assume normal distribution. Moreover, homogenous error variances of control and experimental groups are unlikely – intervention usually transforms error variances in heterogenous ways. All these arguments provide justification for using nonparametric heteroscedastic methods. Heteroscedastic ANOVA-type statistics will be conducted (Brunner et al., 2017) with estimation of relative effects as probabilistic effect size measures. The value of relative effect is the estimation of probability that an observation randomly chosen from the experimental group will have larger value than an observation randomly chosen from the mean distribution function. It follows that any relative effect which statistically significantly differ from 0.50 indicates that an intervention was effective. It was demonstrated that ANOVA-type statistics (ATS) has satisfactory performance for unbalanced groups and small sample sizes (Brunner et al., 2002). Some statistical experts recommend a slightly different version of rank-based non-parametric method, namely the Adjusted Rank Transform Test (ART; see Beasley & Zumbo, 2009; Leys & Schumann, 2010), which is closer to the classical ANOVA (for example, it allows one to compute the generalized eta squared measure, which is routinely used as an effect size indicator). The results of both methods will be reported, with a preliminary note that they converged so there is no need to explain any different outcomes.

Our split-plot design with repeated measures requires to test an interaction between control/experimental groups (factor G) and pretest/posttest time (factor T). We have a control group of participants without intervention (group 1) and an experimental group (group 2) with the active intervention. This design implies that the distribution functions at the start of the experiment (time point 1) are identical because the assignments of participants to the two groups were random. It immediately follows that if the intervention would be effective, it should produce nonparallel time curves of the measurements. We could therefore expect a statistically significant interaction between factor G and factor T. Our hypothesis is that the active intervention is claimed as effective if and only if we reach the statistically significant interaction between groups (control vs. experimental) and time (three time points), not only the significant difference between time points and/or between control and experimental groups. We will not report main factorial effects, because they are of no interest for the purpose of this study.

## Results

To ensure random allocation of participants to groups we conducted preliminary analyses to check for differences between the control and intervention groups on all baseline scores. Since distributions and variances of groups are almost equal, nonparametric Wilcoxon-Mann-Whitney tests were computed and demonstrated that the groups did not differ on any baseline scores (*p-*values ranged between .56 - .97).

### Effect of CMT on Self-Criticism/Reassurance

**Figure 2:**
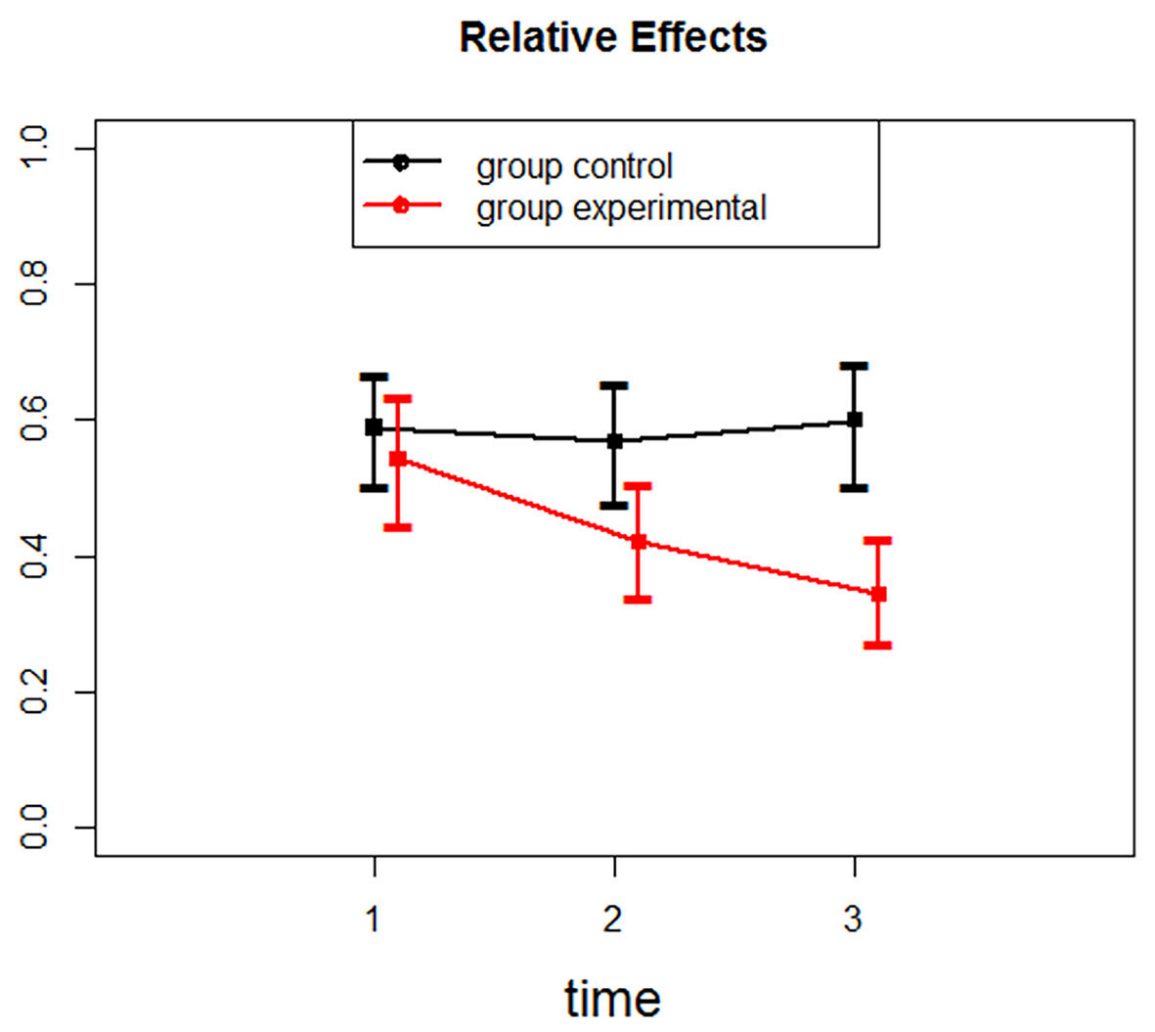
Relative effects for hated-self.

**Figure 3:**
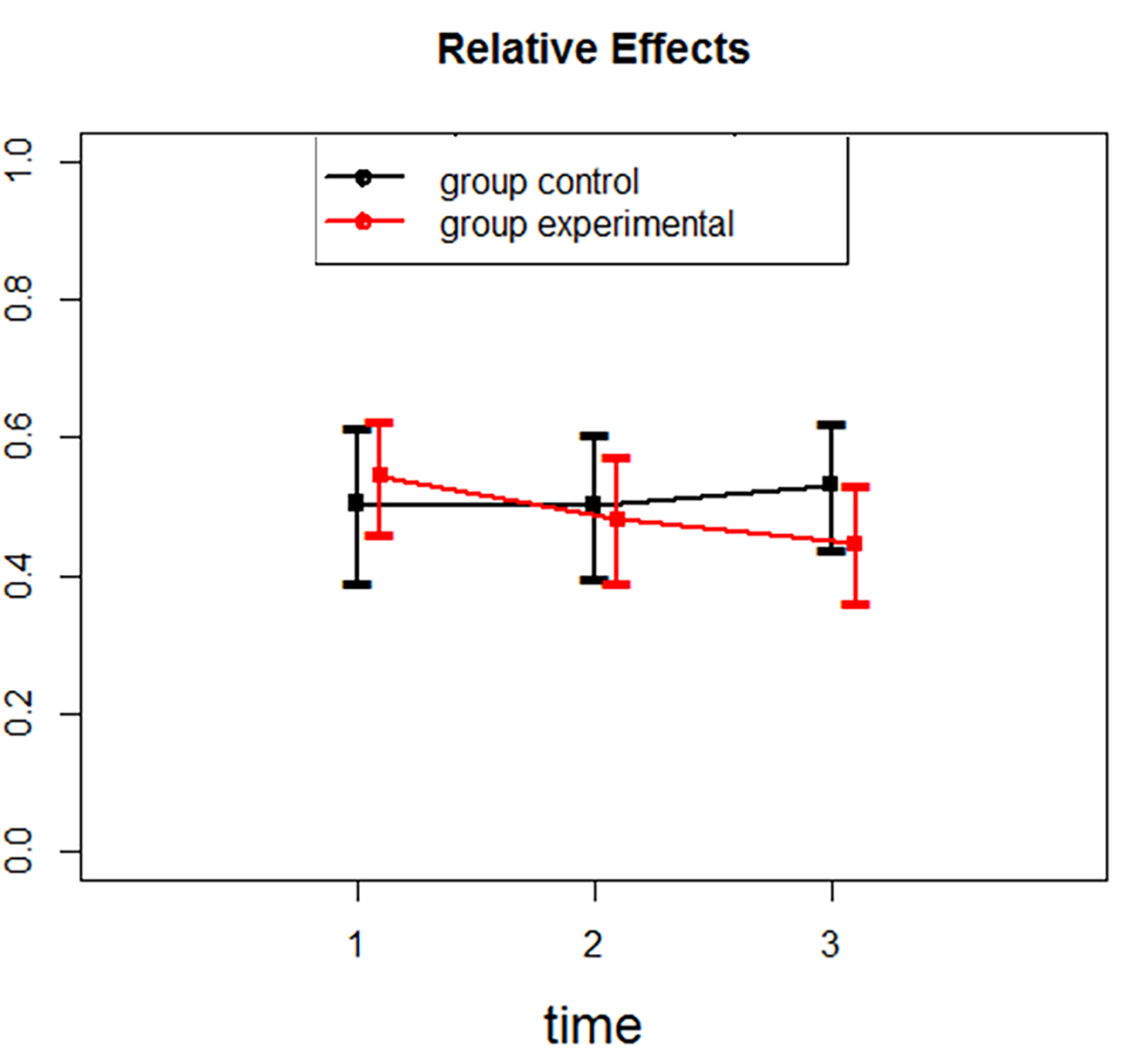
Relative effects for inadequate-self.

A significant effect of the intervention was present on the total score and the self-criticism subscale (combined IS and HS subscales) of the FSCRS and more specifically, the intervention was found to significantly reduce the HS component of the self-criticism scale (see Table 1). There was no effect of the intervention on RS and IS scores. We can inspect in detail relative effects and their confidence intervals for each group and time point (see Table 2): e. g. the relative effects of Hated-Self (see Figure 2) and Inadequate-Self (see Figure 3) demonstrate that there is a statistically significant change in scores of Hated-self but no significant change is present for scores of Inadequate-self.

**Table 1:** Results for Interaction Effects of the FSCRS Scale

FSCRS scores	ATS			ART			
	*F*	*df*	*p*	*F*	*df*	*p*	η_G_^2^
FSCRS total score	7.06	1.74, ∞	.002	5.96	2, 88	.004	.12
FSCRS reassuring	2.12	1.89, ∞	.123	3.19	2, 88	.056	.07
FSCRS inadequate + hated	5.07	1.85, ∞	.008	3.74	2, 88	.028	.08
FSCRS inadequate	2.63	1.72, ∞	.080	1.10	2, 88	.338	.02
FSCRS hated	5.86	1.75, ∞	.004	5.85	2, 88	.005	.12

*Note*. ATS = ANOVA-type statistics; ART = Adjusted Rank Transform Test.

**Table 2:** Relative Effects, Confidence Intervals and Variances of the FSCRS Scale

Group / Time	Relative effect	Confidence interval	Variance
	FSCRS total score		
Control			
Pretest	.54	.45 – .63	0.108
Posttest	.52	.41 – .62	0.131
Follow-up	.53	.43 – .63	0.122
Intervention			
Pretest	.56	.47 – .65	0.092
Posttest	.46^a^	.38 – .49	0.082
Follow-up	.38^a^	.31 – .47	0.081
	FSCRS reassured-self		
Control			
Pretest	.52	.41 – .62	0.135
Posttest	.51	.40 – .62	0.152
Follow-up	.53	.41 – .63	0.154
Intervention			
Pretest	.57	.48 – .65	0.129
Posttest	.44	.36 – .53	0.088
Follow-up	.46	.38 – .54	0.080
	FSCRS inadequate + hated-self		
Control			
Pretest	.53	.43 – .63	0.102
Posttest	.51	.41 – .61	0.121
Follow-up	.51	.42 – .60	0.126
Intervention			
Pretest	.54	.44 – .62	0.097
Posttest	.46	.38 – .55	0.092
Follow-up	.41^a^	.33 – .49	0.083
	FSCRS inadequate-self		
Control			
Pretest	.51	.40 – .61	0.101
Posttest	.50	.39 – .62	0.139
Follow-up	.53	.43 – .62	0.160
Intervention			
Pretest	.54	.46 – .62	0.102
Posttest	.48	.39 – .57	0.089
Follow-up	.44	.36 – .53	0.082
	FSCRS hated-self		
Control			
Pretest	.57	.48 – .65	0.100
Posttest	.59	.50 – .67	0.100
Follow-up	.60^a^	.50 – .68	0.080
Intervention			
Pretest	.54	.44 – .64	0.114
Posttest	.42^a^	.34 – .49	0.086
Follow-up	.34^a^	.27 – .43	0.072

^a^The effectivity of an intervention is indicated by relative effect significantly higher (for increasing effect) or lower (for decreasing effect) than 0.50.

### Effect of CMT on Self-Compassion

A significant effect of the intervention on the total SCS score was found with this effect specifically evident on combined scores from the self-judgement, isolation and over-identification subscales (Table 3). The intervention had no effect on kindness, common humanity and mindfulness subscale scores which represent the positively formulated items of self-compassion. Again, relative effects with their confidence intervals for each group and time point (Table 4) enable to view the effect in more detail.

**Table 3:** Results for Interaction Effects of the SCS Scale

SCS scores	ATS			ART			
	*F*	*df*	*p*	*F*	*df*	*p*	η_G_^2^
SCS sum score	5.17	1.82, ∞	.006	6.67	2, 88	.003	0.13
SCS positive	2.34	1.97, ∞	.098	2.92	2, 88	.059	0.06
SCS negative	4.32	1.85, ∞	.016	4.32	2, 88	.017	0.09

*Note*. ATS = ANOVA-type statistics; ART = Adjusted Rank Transform Test.

**Table 4:** Relative Effects, Their Confidence Intervals and Variances of the SCS Scale

Group / Time	Relative effect	Confidence interval	Variance
	SCS sum score		
Control			
Pretest	.54	.45 – .63	0.108
Posttest	.52	.41 – .62	0.131
Follow-up	.53	.43 – .63	0.122
Intervention			
Pretest	.56	.47 – .65	0.092
Posttest	.46^a^	.38 – .49	0.082
Follow-up	.38^a^	.31 – .47	0.081
	SCS positive		
Control			
Pretest	.52	.41 – .62	0.135
Posttest	.51	.40 – .62	0.152
Follow-up	.53	.41 – .63	0.154
Intervention			
Pretest	.57	.48 – .65	0.129
Posttest	.44	.36 – .53	0.088
Followup	.46	.38 – .54	0.080
	SCS negative		
Control			
Pretest	.53	.43 – .63	0.102
Posttest	.51	.41 – .61	0.121
Follow-up	.51	.42 – .60	0.126
Intervention			
Pretest	.54	.44 – .62	0.097
Posttest	.46	.38 – .55	0.092
Follow-up	.41^a^	.33 – .49	0.083

^a^Statistically significant at .05 level.

## Discussion

The present study assessed the impact and longer term effects of a 13-day online version of the Compassion Mind Training on perceived levels of self-compassion and self-criticism. to the best of our knowledge, this is the first study to test the effect of CMT when delivered as an online-based intervention to a nonclinical sample.

The findings demonstrate that CMT reduced negative thoughts and feelings specifically relating to hostility towards oneself as measured by the Hated-self subscale of the FSCRS (Gilbert et al., 2004) and components of self-compassion relating to self-judgement, isolation and over-identification (Neff, 2003).

Our study supports previous findings that CMT results in a reduction in self-criticism in a nonclinical sample (Matos et al., 2017) and that CMT influences changes to Hated-self but not Inadequate-self (Judge et al., 2012; Lucre & Corten, 2013). These findings are also consonant with Gilbert (2010b) who distinguished Inadequate-Self as a corrective function and Hated-Self as a pathological attitude towards oneself.

The current findings also indicate that whilst CMT may reduce the negative components of self-compassion, it highlights that it does not improve self-compassion, which is contrast in to Mindful Self-compassion Training (Neff & Germer, 2013) which has been found to improve self-compassion (Halamová, Kanovský, Jakubcová, & Kupeli, 2020). These results support the idea that self-compassion and self-criticism are not structured as a dichotomous construct and therefore should not be used as opposites. Yet, it implies that there may be benefit in combining these two approaches. However, this requires further research which is beyond the present study.

Recent psychometric evaluation of the FSCRS showed that the scores from the three subscales can be used in either as a total score or as two factors consisting of Self-Reassurance and Self-Criticism or as three factors of Hated-Self, Inadequate-Self and Reassured-Self (Halamová, Kanovský, & Pacúchová, 2017). Although, a two-factor model of Self-Reassurance and Self-Criticism (by merging Hated-Self and Inadequate-Self) showed the best fit (Halamová, Kanovský, & Pacúchová, 2017), the current study highlights that Hated-self and Inadequate-self are distinct forms of Self-criticism and should be examined independently (Judge et al., 2012; Lucre & Corten, 2013).

These findings also support the idea that a combination of the SCS may not be suitable for evaluating the efficacy of interventions. A recent international comparison of the factor structure of the SCS suggests that Self-compassionate responding (positive subscales of the SCS) and Self-uncompassionate responding (negative subscales of the SCS) are related, but are still different psychological constructs and so therefore should be measured separately (Brenner, Heath, Vogel, & Credé, 2017; López et al., 2015). In our study, the overall SCS score increased significantly but only due to a reduction in Self-uncompassionate responding. In summary, using the total SCS score to assess the effect of an intervention may not distinguish if increases in the total SCS score are due to improvements to self-compassion or a reduction in self-uncompassionate responding. Therefore, it is recommended that the subscales of the SCS should not be combined when assessing the effectiveness of interventions and treatments (Brenner, Heath, Vogel, & Credé, 2017; López et al., 2015).

Interestingly, the control group reported a significant increase in their Hated-self score which is suggestive of the sensitisation effect. Completing these measures three times may have resulted in more thoughts about the hostility they project on themselves and thus result in an increase in self-criticism.

Although CMT was designed as a group-based therapy (Gilbert & Irons, 2004), our research shows that it could successfully be implemented in an online, individual format with beneficial effects in a non-clinical population. These findings are promising as interventions can be developed using a similar easy-to-administer format to target wider populations and to determine how self-criticism can be treated in the most efficient form.

Our findings cannot be generalised to a clinical sample and future research should replicate the present study with a clinical sample to explore if an online-based CMT intervention is effective in reducing clinical levels psychopathology. It would also be beneficial to explore if an online-based psychological intervention of this type would be feasible and acceptable for people across clinical samples.

To date, the majority of studies implementing CMT have been conducted in English in the UK. So far, two published studies have explored the utility of the CMT outside of the UK (Portugal [Matos et al., 2017]; Iran [Noorbala et al., 2013]). Therefore, the current study is one of few studies which have explored the impact of CMT in a different language (Slovak) and culture and contributes to the cross-cultural credibility of the CMT.

### Strengths and Limitations

The current study did not collect data on participant’s psychological wellbeing both before undertaking the intervention and during, thus making it possible that the sample consisted of people with a clinical diagnosis of psychopathology.

Due to the level of commitment required to complete the intervention tasks and high attrition rate, it is possible that only those who were highly motivated or interested in the topic area remained in the study. However, this was not surprising as online-based interventions have been found to be at high risk of attrition (Eysenbach, 2005) but this study has demonstrated that an online version of CMT is acceptable and feasible to implement with minimal facilitation.

## Conclusion

An abbreviated, online version of the Compassion Mind Training has significantly reduced self-criticism, in particular thoughts and feelings of hostility as measured by the Hated-Self, subscale of the FSCRS and Self-uncompassionate responding as measured by the SCS. In addition, these changes were still evident after two months. The results are promising and posit that interventions can be developed using virtual technology to reach wider populations without direct involvement of mental health professionals. These findings are particularly valuable relevant for those who might experience stigma as a result of mental health or be unable to contact a mental health care provider.

## Data Availability

In order to comply with the ethics approvals of the study protocols, data cannot be made accessible through a public repository. However, data are available upon request for researchers who consent to adhering to the ethical regulations for confidential data.

## Appendix 1

**Table A1:** Description of Previous Research Measuring Effectiveness of CMT on the Level of Self-Criticism and Self-Compassion

Study (country)	Participants (*n*)	Control / follow up	Sessions	Measure(s)	Significant outcomes
(Gilbert & Irons, 2004), UK	Depression (8)	No/No	Group sessions for 1.5 hours once a week over four weeks	Quantitative ratings of self-criticism and self-compassion in diaries	Increase in ease of generating self-compassionate images and self-soothing in self-critical situations
(Gilbert & Procter, 2006), UK	Personality disorders and/or mood disorders (6)	No/No	Two-hour group sessions a week over 12 weeks	FSCRS, FSCS	Reduction in self-criticism, IS and HS; increases in RS
(Laithwaite et al., 2009), UK	Psychosis (18)	No/Yes: Six weeks	20 group sessions over 10 weeks	SCS	No change in self-compassion
(Beaumont et al., 2012), UK	Trauma (16)	No/No	Weekly group meetings for up to 12 weeks	SCS-SF	Increase in self-compassion
(Braehler et al., 2013), UK	Psychosis (22)	Yes/No	Two-hour group sessions per week over 16 weeks	Narrative Recovery Style Scale (Gumley et al., 2010)	More compassion in narratives
(Judge et al., 2012), UK	Heterogeneous diagnosis (27)	No/No	Two-hour group sessions over 12-14 weeks	FSCRS, FSCS	Increase in RS and reduction in HS, IS
(Noorbala et al., 2013), Iran	Major Depressive Disorder (19)	Yes/Yes: Two months	Two-hour group sessions twice weekly over 6 weeks	LOSC	No change in self-criticism
(Lucre & Corten, 2013), UK	Personality disorder (8)	No/Yes: One year	Group meetings over 16 weeks	FSCRS	Increase in RS and reduction in HS
(Beaumont et al., 2016), UK	Non-clinical (28)	No/No	Three-day group training	SCS-SF, FSCS	Increase in self-compassion and reduction in Self-uncompassionate responding
(Matos et al., 2017), Portugal	Non-clinical (56)	Yes/No	Two-hour group sessions at the beginning and at the end and two weeks of individual practicing	FSCRS, SCS	Changes in Self-Compassion, Self-Criticism, HS, IS and RS
The present study	Non-clinical (26)	Yes/Yes: Two months	Individual daily exercise for two weeks provided in an online format	FSCRS, SCS	Changes in HS and Self-uncompassionate responding

*Note*. FSCRS = Forms of Self-criticism/Attacking & Self-Reassuring Scale (Gilbert et al., 2004); FSCS = Functions of Self-criticizing (Gilbert et al., 2004) HS = Hated-self; IS = Inadequate-self; LOSC = Levels of Self-Criticism Scale (Thompson & Zuroff, 2004); RS = Reassured-self; SCS = Self-Compassion Scale (Neff, 2003); SCS-SF = Self-Compassion Scale Short Form (Raes et al., 2011).

## Appendix 2

**Table A2:** Description of Selected Exercises Included in the Short-Term Online CMT Intervention

Exercise	Description of the exercise
Soothing Rhythm Breathing	This exercise gently focuses on deep diaphragm breathing and finding a breathing rhythm which is personally soothing, comforting and calming.
Compassionate Body Scan	During this exercise one gradually concentrates on different parts of body from legs to head whilst imagining release of tension and relaxing the body.
Imaging the Self-Critical part of Self	Imagining own self-critical part as a person with specific appearance, nonverbal, paraverbal, and verbal communication and with a particular relationship to the participant.
Creating a Safe Place	During this exercise the individual is encouraged to create a safe place in his/her imagination with specific sensations: seeing, hearing, touching, tasting or smelling and enjoying being there.
Compassionate Colour	This guided imagery exercise involves imagining a colour which one can associate with compassion, a colour which conveys warmth and kindness, support and strength.
Developing the Inner Compassionate Self	This exercise is about imagining yourself as a compassionate person, being able to think, act and feel compassionately and having qualities such as wisdom, responsibility, warmth, and strength.
Working with Troubled Self	Imagine watching a film of yourself while holding compassionate stance. Compassionately see yourself how you get through the day and compassionately notice how you are troubled by self-critical feelings or thoughts.
Working with Anxious Self	Imagine watching a film with yourself while being compassionate. Compassionately see how many things during the day make you anxious and feel compassion for the anxiety and help to reduce anxiety.
Compassion Flowing into you from Others	This exercise encourages individuals to recall a memory when somebody was kind to them. They are guided to remember the person his/her appearance, tone of voice, what he/she said and focus on the feeling they experienced when they received compassion from that person.
Compassion Flowing Out	Imagine kindness flowing from you to others by recalling a memory when you felt kindness towards someone else. Imagine pleasure of being kind to somebody else.
Compassionate Letter Writing	During this exercise one writes a letter from own compassionate part to yourself having troubles. While writing, it is important to create as much warmth and understanding as possible.
Compassionate Dialogue Writing	Imagine dialogue with your compassionate Self, a Self that understands you completely and it is caring and accepting. Imagine what would you say to each other and what would you feel during the dialogue etc.
Creating your Ideal Caring-compassionate Image	Imagine creating and meeting your compassionate ideal image, its appearance, various sensations of it, relationship with it while remembering that the image values you and cares about you unconditionally wishing you happiness and free of suffering.

*Note*. The exercises were selected from a multitude of different exercises available from Gilbert (2010a).
